# 5-Ethyl-5-methyl-4-phenyl-5*H*-1,2,4-triazol-3(4*H*)-thione

**DOI:** 10.1107/S1600536810030503

**Published:** 2010-08-11

**Authors:** Kong Wai Tan, M. Jamil Maah, Seik Weng Ng

**Affiliations:** a Department of Chemistry, University of Malaya, 50603 Kuala Lumpur, Malaysia

## Abstract

The five-membered ring of the title compound Δ^1^-1,2,4-triazoline-5-thione, C_11_H_13_N_3_S, is almost planar (r.m.s. deviation = 0.009 Å); the phenyl ring is aligned at 84.6 (2)° with respect to the five-membered ring. The crystal studied was a racemic twin with an approximate 20% minor twin component. Weak inter­molecular C—H⋯N hydrogen bonding is present in the crystal structure.

## Related literature

For the synthesis of this and other Δ^1^-[1,2,4]-triazoline-5-thio­nes, see: Kabashima *et al.* (1991 ▶); Landquist (1970 ▶); Tripathi & Dhar (1986 ▶). For the crystal structure of the related compound 5,5-dimethyl-4-phenyl-1,2,4-triazol-3-thione, see: Katritzky *et al.* (1984 ▶).
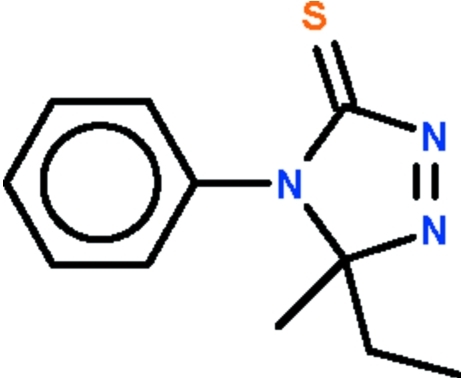


## Experimental

### 

#### Crystal data

C_11_H_13_N_3_S
*M* *_r_* = 219.30
Tetragonal, 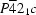

*a* = 17.962 (4) Å
*c* = 6.9992 (14) Å
*V* = 2258.2 (6) Å^3^
*Z* = 8
Mo *K*α radiation
μ = 0.26 mm^−1^
*T* = 100 K
0.30 × 0.05 × 0.05 mm

#### Data collection

Bruker SMART APEX diffractometer
Absorption correction: multi-scan (*SADABS*; Sheldrick, 1996 ▶) *T*_min_ = 0.927, *T*_max_ = 0.987
10418 measured reflections
1987 independent reflections
1546 reflections with *I* > 2σ(*I*)
*R*_int_ = 0.087

#### Refinement

*R*[*F* ^2^ > 2σ(*F* ^2^)] = 0.065
*wR*(*F* ^2^) = 0.186
*S* = 1.07
1987 reflections
136 parameters
H-atom parameters constrained
Δρ_max_ = 0.69 e Å^−3^
Δρ_min_ = −0.31 e Å^−3^
Absolute structure: Flack (1983 ▶), 837 Friedel pairs
Flack parameter: −0.2 (2)

### 

Data collection: *APEX2* (Bruker, 2009 ▶); cell refinement: *SAINT* (Bruker, 2009 ▶); data reduction: *SAINT*; program(s) used to solve structure: *SHELXS97* (Sheldrick, 2008 ▶); program(s) used to refine structure: *SHELXL97* (Sheldrick, 2008 ▶); molecular graphics: *X-SEED* (Barbour, 2001 ▶); software used to prepare material for publication: *publCIF* (Westrip, 2010 ▶).

## Supplementary Material

Crystal structure: contains datablocks global, I. DOI: 10.1107/S1600536810030503/xu5008sup1.cif

Structure factors: contains datablocks I. DOI: 10.1107/S1600536810030503/xu5008Isup2.hkl

Additional supplementary materials:  crystallographic information; 3D view; checkCIF report

## Figures and Tables

**Table 1:** Hydrogen-bond geometry (Å, °)

*D*—H⋯*A*	*D*—H	H⋯*A*	*D*⋯*A*	*D*—H⋯*A*
C9—H9*A*⋯N2^i^	0.98	2.56	3.519 (9)	165

Symmetry code: (i) 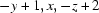.

**Fig. 1.**
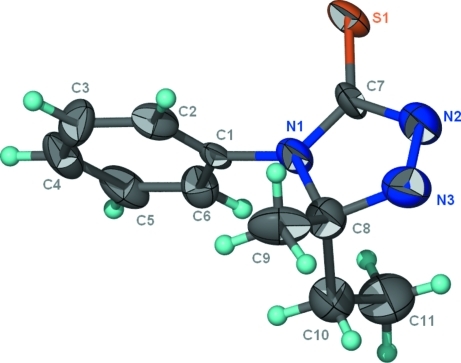
Thermal ellipsoid plot (Barbour, 2001) of C11H13N3S at the 70% probability level; hydrogen atoms are drawn as spheres of arbitrary radius.

## supplementary crystallographic information

### Comment

3-Phenyl-Δ^1^-[1,2,4]-triazoline-5-thiones are synthesized by the heterocyclization of the Schiff base condensation product of the reaction between phenylthiosemicarbazide and a ketone in the presence of chlorocarbonylsulfenyl chloride (Kabashima *et al.*, 1991), chlorosulfonyl isocyanate (Tripathi & Dhar, 1986) and manganese dioxide (Landquist, 1970). In the present study, the oxidizing agent is 1,10-phenanthroline-5,6-dione, commonly known as phendione. 4-Phenyl thiosemicarbazide condensed with methyl ethyl ketone to form the initial Schiff base, which was then oxidized to the title compound by phendione (Scheme I, Fig. 1). Intermolecular weak C—H···N hydrogen bonding is present in the crystal structure (Table 1).

### Experimental

4-Phenyl thiosemicarbazide (2 mmol, 0.33 g) and 1,10-phenanthroline-5,6-dione (1 mmol, 0.21 g) were heated in a mixture of methyl ethyl ketone (5 ml) and ethanol (10 ml). The yellow precipitate that formed was removed by filtration. Slow evaporation of the orange filtrate afforded the title compound.

### Refinement

Carbon-bound H-atoms were placed in calculated positions (C—H 0.95–0.99 Å) and were included in the refinement in the riding model approximation, with *U*(H) set to 1.2–1.5*U*(C).

### Crystal data

**Table:**

C_11_H_13_N_3_S	*D*_x_ = 1.290 Mg m^−^^3^
*M**_r_* = 219.30	Mo *K*α radiation, λ = 0.71073 Å
Tetragonal, *P*42_1_*c*	Cell parameters from 926 reflections
Hall symbol: P -4 2n	θ = 2.5–18.5°
*a* = 17.962 (4) Å	µ = 0.26 mm^−^^1^
*c* = 6.9992 (14) Å	*T* = 100 K
*V* = 2258.2 (6) Å^3^	Prism, orange
*Z* = 8	0.30 × 0.05 × 0.05 mm
*F*(000) = 928	

### Data collection

**Table:**

Bruker SMART APEX diffractometer	1987 independent reflections
Radiation source: fine-focus sealed tube	1546 reflections with *I* > 2σ(*I*)
graphite	*R*_int_ = 0.087
ω scans	θ_max_ = 25.0°, θ_min_ = 2.3°
Absorption correction: multi-scan (*SADABS*; Sheldrick, 1996)	*h* = −21→20
*T*_min_ = 0.927, *T*_max_ = 0.987	*k* = −21→21
10418 measured reflections	*l* = −8→5

### Refinement

**Table:**

Refinement on *F*^2^	Secondary atom site location: difference Fourier map
Least-squares matrix: full	Hydrogen site location: inferred from neighbouring sites
*R*[*F*^2^ > 2σ(*F*^2^)] = 0.065	H-atom parameters constrained
*wR*(*F*^2^) = 0.186	*w* = 1/[σ^2^(*F*_o_^2^) + (0.0992*P*)^2^ + 1.8759*P*] where *P* = (*F*_o_^2^ + 2*F*_c_^2^)/3
*S* = 1.07	(Δ/σ)_max_ = 0.001
1987 reflections	Δρ_max_ = 0.69 e Å^−^^3^
136 parameters	Δρ_min_ = −0.31 e Å^−^^3^
0 restraints	Absolute structure: Flack (1983), 837 Friedel pairs
Primary atom site location: structure-invariant direct methods	Flack parameter: −0.2 (2)

### Fractional atomic coordinates and isotropic or equivalent isotropic displacement parameters (Å^2^)

**Table:**

	*x*	*y*	*z*	*U*_iso_*/*U*_eq_	
S1	0.47452 (8)	0.23341 (7)	1.2411 (2)	0.0351 (4)	
N1	0.4463 (3)	0.2810 (3)	0.8841 (6)	0.0351 (11)	
N2	0.4094 (3)	0.3589 (3)	1.1143 (7)	0.0531 (15)	
N3	0.3934 (3)	0.3924 (3)	0.9623 (8)	0.0511 (14)	
C1	0.4778 (3)	0.2198 (2)	0.7806 (6)	0.0237 (10)	
C2	0.5518 (3)	0.2275 (3)	0.7230 (8)	0.0353 (13)	
H2	0.5801	0.2706	0.7530	0.042*	
C3	0.5823 (3)	0.1677 (3)	0.6175 (9)	0.0441 (15)	
H3	0.6323	0.1704	0.5738	0.053*	
C4	0.5409 (4)	0.1065 (3)	0.5785 (8)	0.0480 (17)	
H4	0.5623	0.0671	0.5066	0.058*	
C5	0.4687 (4)	0.1003 (3)	0.6404 (8)	0.0435 (15)	
H5	0.4406	0.0568	0.6136	0.052*	
C6	0.4381 (3)	0.1576 (3)	0.7413 (9)	0.0351 (12)	
H6	0.3881	0.1537	0.7846	0.042*	
C7	0.4431 (3)	0.2869 (3)	1.0741 (7)	0.0371 (14)	
C8	0.4127 (3)	0.3477 (3)	0.7940 (8)	0.0398 (14)	
C9	0.4652 (4)	0.3911 (3)	0.6695 (9)	0.0525 (17)	
H9A	0.5098	0.4041	0.7429	0.079*	
H9B	0.4407	0.4367	0.6254	0.079*	
H9C	0.4794	0.3607	0.5590	0.079*	
C10	0.3418 (3)	0.3257 (4)	0.6851 (9)	0.0528 (18)	
H10A	0.3210	0.3707	0.6231	0.063*	
H10B	0.3554	0.2902	0.5828	0.063*	
C11	0.2824 (4)	0.2911 (5)	0.8070 (11)	0.073 (2)	
H11A	0.2391	0.2789	0.7276	0.109*	
H11B	0.2675	0.3262	0.9072	0.109*	
H11C	0.3017	0.2455	0.8659	0.109*	

### Atomic displacement parameters (Å^2^)

**Table:**

	*U*^11^	*U*^22^	*U*^33^	*U*^12^	*U*^13^	*U*^23^
S1	0.0531 (8)	0.0347 (6)	0.0174 (6)	−0.0009 (6)	−0.0038 (7)	0.0016 (7)
N1	0.051 (3)	0.033 (2)	0.021 (2)	0.014 (2)	−0.004 (2)	−0.001 (2)
N2	0.069 (4)	0.058 (3)	0.033 (3)	0.025 (3)	−0.008 (3)	−0.007 (3)
N3	0.058 (3)	0.048 (3)	0.047 (3)	0.010 (3)	−0.015 (3)	−0.009 (3)
C1	0.035 (2)	0.024 (2)	0.012 (2)	0.0085 (19)	−0.006 (2)	0.000 (2)
C2	0.042 (3)	0.031 (3)	0.032 (3)	−0.009 (2)	−0.009 (3)	0.000 (3)
C3	0.036 (3)	0.055 (4)	0.041 (4)	0.012 (3)	0.015 (3)	0.008 (3)
C4	0.088 (5)	0.034 (3)	0.022 (3)	0.023 (3)	0.001 (3)	0.002 (3)
C5	0.072 (4)	0.029 (3)	0.029 (3)	−0.006 (3)	−0.010 (3)	−0.003 (2)
C6	0.038 (3)	0.038 (3)	0.030 (3)	−0.001 (2)	0.006 (3)	0.001 (3)
C7	0.053 (4)	0.042 (3)	0.016 (3)	0.019 (2)	−0.002 (2)	−0.008 (2)
C8	0.044 (3)	0.039 (3)	0.037 (4)	0.006 (2)	−0.001 (3)	−0.001 (3)
C9	0.059 (4)	0.048 (3)	0.051 (4)	−0.006 (3)	−0.019 (3)	0.021 (3)
C10	0.047 (4)	0.066 (4)	0.046 (4)	0.004 (3)	−0.003 (3)	−0.009 (3)
C11	0.045 (4)	0.101 (6)	0.072 (6)	−0.004 (4)	−0.009 (4)	−0.012 (5)

### Geometric parameters (Å, °)

**Table:**

S1—C7	1.614 (5)	C5—C6	1.364 (8)
N1—C7	1.335 (7)	C5—H5	0.9500
N1—C1	1.433 (6)	C6—H6	0.9500
N1—C8	1.483 (7)	C8—C9	1.502 (8)
N2—N3	1.255 (7)	C8—C10	1.535 (8)
N2—C7	1.456 (7)	C9—H9A	0.9800
N3—C8	1.466 (8)	C9—H9B	0.9800
C1—C6	1.355 (7)	C9—H9C	0.9800
C1—C2	1.396 (7)	C10—C11	1.502 (10)
C2—C3	1.415 (8)	C10—H10A	0.9900
C2—H2	0.9500	C10—H10B	0.9900
C3—C4	1.355 (9)	C11—H11A	0.9800
C3—H3	0.9500	C11—H11B	0.9800
C4—C5	1.372 (9)	C11—H11C	0.9800
C4—H4	0.9500		
			
C7—N1—C1	125.5 (5)	N2—C7—S1	122.3 (4)
C7—N1—C8	110.0 (5)	N3—C8—N1	101.3 (4)
C1—N1—C8	124.5 (4)	N3—C8—C9	109.3 (5)
N3—N2—C7	110.9 (5)	N1—C8—C9	114.2 (5)
N2—N3—C8	111.4 (4)	N3—C8—C10	110.1 (5)
C6—C1—C2	121.7 (4)	N1—C8—C10	109.9 (5)
C6—C1—N1	121.8 (5)	C9—C8—C10	111.5 (5)
C2—C1—N1	116.5 (4)	C8—C9—H9A	109.5
C1—C2—C3	116.4 (5)	C8—C9—H9B	109.5
C1—C2—H2	121.8	H9A—C9—H9B	109.5
C3—C2—H2	121.8	C8—C9—H9C	109.5
C4—C3—C2	120.6 (5)	H9A—C9—H9C	109.5
C4—C3—H3	119.7	H9B—C9—H9C	109.5
C2—C3—H3	119.7	C11—C10—C8	114.4 (6)
C3—C4—C5	121.4 (5)	C11—C10—H10A	108.7
C3—C4—H4	119.3	C8—C10—H10A	108.7
C5—C4—H4	119.3	C11—C10—H10B	108.7
C6—C5—C4	119.0 (5)	C8—C10—H10B	108.7
C6—C5—H5	120.5	H10A—C10—H10B	107.6
C4—C5—H5	120.5	C10—C11—H11A	109.5
C1—C6—C5	121.0 (5)	C10—C11—H11B	109.5
C1—C6—H6	119.5	H11A—C11—H11B	109.5
C5—C6—H6	119.5	C10—C11—H11C	109.5
N1—C7—N2	106.3 (5)	H11A—C11—H11C	109.5
N1—C7—S1	131.2 (5)	H11B—C11—H11C	109.5
			
C7—N2—N3—C8	0.9 (7)	C8—N1—C7—S1	−176.6 (5)
C7—N1—C1—C6	85.0 (7)	N3—N2—C7—N1	0.5 (7)
C8—N1—C1—C6	−95.6 (6)	N3—N2—C7—S1	175.9 (5)
C7—N1—C1—C2	−94.8 (7)	N2—N3—C8—N1	−1.9 (6)
C8—N1—C1—C2	84.7 (6)	N2—N3—C8—C9	−122.7 (6)
C6—C1—C2—C3	1.5 (7)	N2—N3—C8—C10	114.5 (6)
N1—C1—C2—C3	−178.8 (4)	C7—N1—C8—N3	2.2 (6)
C1—C2—C3—C4	−0.7 (8)	C1—N1—C8—N3	−177.3 (5)
C2—C3—C4—C5	−0.5 (9)	C7—N1—C8—C9	119.6 (6)
C3—C4—C5—C6	0.9 (9)	C1—N1—C8—C9	−60.0 (7)
C2—C1—C6—C5	−1.1 (8)	C7—N1—C8—C10	−114.3 (5)
N1—C1—C6—C5	179.1 (5)	C1—N1—C8—C10	66.2 (7)
C4—C5—C6—C1	−0.1 (9)	N3—C8—C10—C11	−50.8 (8)
C1—N1—C7—N2	177.8 (5)	N1—C8—C10—C11	60.0 (7)
C8—N1—C7—N2	−1.8 (7)	C9—C8—C10—C11	−172.4 (6)
C1—N1—C7—S1	2.9 (10)		

### Hydrogen-bond geometry (Å, °)

**Table:**

*D*—H···*A*	*D*—H	H···*A*	*D*···*A*	*D*—H···*A*
C9—H9A···N2^i^	0.98	2.56	3.519 (9)	165

Symmetry codes: (i) −*y*+1, *x*, −*z*+2.
